# Decreasing Seroma Incidence Following Abdominoplasty: A Systematic Review and Meta-Analysis of High-Quality Evidence

**DOI:** 10.1093/asjof/ojae016

**Published:** 2024-03-16

**Authors:** Christopher D Liao, Kelley Zhao, Nia Nikkhahmanesh, Duc T Bui

## Abstract

**Background:**

Seroma formation is the most common complication of abdominoplasties. Many preventive interventions have been proposed, but none have been recognized as a definitive solution, partly due to varying levels of evidence (LOE) in the literature.

**Objectives:**

We aimed to analyze seroma prevention methods supported by high-level evidence.

**Methods:**

The PubMed database was queried through August 2023. Primary articles of interest included randomized controlled trials (RCTs), prospective comparative studies, and meta-analyses of these studies. The LOE for each article was determined according to the American Society of Plastic Surgeons Rating Scale. The “seroma occurrence ratio,” or ratio of seroma events in the interventional group to respective control group, was calculated to compare incidence rates between techniques.

**Results:**

Twenty articles and 9 categories of techniques were analyzed. Study designs included 10 RCTs, 2 prospective cohort studies, 7 prospective comparative studies, and 1 retrospective randomized study. The use of progressive-tension and quilting sutures had the most data supporting a statistically significant reduction in seroma (occurrence ratio 0.306, *P* < .001). Tissue adhesives and preservation of Scarpa's fascia were also well reinforced (0.375, *P* < .01 and 0.229, *P* < .011, respectively), while increasing the number of drains was not (*P* = .7576). Meta-analysis demonstrated that compared with 2 drains alone, alternative techniques were more effective at reducing seroma occurrence (pooled risk ratio 0.33, 95% CI, 0.11-0.99).

**Conclusions:**

This review highlights multiple seroma prevention techniques for abdominoplasty investigated in recent high-quality literature. We suggest future randomized comparative studies of the various seroma prevention methods to fully ascertain their efficacy following abdominoplasty.

**Level of Evidence: 1:**

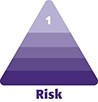

Abdominoplasty is one of the most commonly performed operations by plastic surgeons.^1^ Among complications, such as bleeding, tissue necrosis, or infection,^2^ one particular consequence of the wide area of dissection involved is an increased risk of fluid accumulation. Therefore, seroma remains the most common complication of abdominoplasty.^3-7^ Although the acceptable standard for seroma formation is 10%,^8^ incidence rates of up to 25% have been reported.^2^ Several mechanisms have been implicated, including disruption of lymphatic and vascular ducts,^2^ dead space formation,^5^ and shearing forces, causing release of inflammatory mediators.^9^ Although often self-limited, untreated seromas can cause wound dehiscence, infection, flap necrosis, and pseudocyst formation, resulting in poor cosmetic results and prolonged hospitalization.^7,10^ Given that abdominoplasties are generally elective operations, it is imperative to minimize the likelihood of seroma formation.^11^

Many interventions have been proposed to prevent seroma formation following abdominoplasties, including suction drains,^12^ fibrin sealants,^13^ quilting sutures (QSs),^3^ progressive tension sutures (PTSs),^14^ and preservation of Scarpa's fascia.^15^ Of these interventions, the most commonly applied are postoperative drains implemented by 98% of plastic surgeons after abdominoplasties.^16^ Despite numerous studies examining these interventions, none have been recognized as the optimal method for seroma prevention.^10,17^ Due to sparse literature comparing the performance of these interventions with one another, there remains a broad spectrum of practices to reduce seroma formation.

Although a systematic review of prospective randomized controlled trials (RCTs) was published by Seretis et al,^18^ the study's strict selection criteria excluded papers and other study designs that still demonstrated a high level of evidence (LOE) and could therefore contribute additional high-quality data. Given the body of literature added since then as well as the varying LOEs, there is a continued need for an updated and more comprehensive review of high-quality publications for minimizing the risk of seroma occurrence following abdominoplasty. Therefore, the objective of this study is to analyze all currently available high-LOE publications on seroma prevention methods and compare their efficacies.

## METHODS

This study adhered to the Preferred Reporting Items for Systematic Reviews and Meta-Analyses guidelines. The PubMed database was queried for all full-text, English publications using the following search terms: (“Abdominoplasty”[Mesh] OR abdominoplasty) AND (“Surgical Wound Infection”[Mesh] OR “surgical wound infection” OR “surgical site infection” OR “Seroma”[Mesh] OR seroma). The term “infection” was incorporated as a search term to ensure that the search would return any potential studies that may have included infections secondary to seroma formation. A total of 355 results were returned on June 16, 2021 with no date restrictions. A total of 89 additional studies were returned after an updated literature search on August 23, 2023 with date restrictions from June 16, 2021 to August 23, 2023. Titles and abstracts were reviewed for relevance to interventions intended to reduce seroma occurrence following abdominoplasty. Primary articles of interest included high-quality study designs, such as RCTs, prospective comparative studies, and meta-analyses of these studies. Selected meta-analyses were reviewed for additional citations that were not retrieved upon initial query; these individual studies were included for analysis instead of the meta-analyses themselves to prevent redundancy. Retrospective studies were included only if randomized study designs were utilized. All other retrospective studies, prospective cohort studies without a comparison group, case series, and animal studies were excluded. Articles describing operations such as hernia repair, panniculectomies, abdominal wall reconstruction, and abdominal closures performed for deep inferior epigastric perforator flap harvest were also excluded.

**Figure 1.**
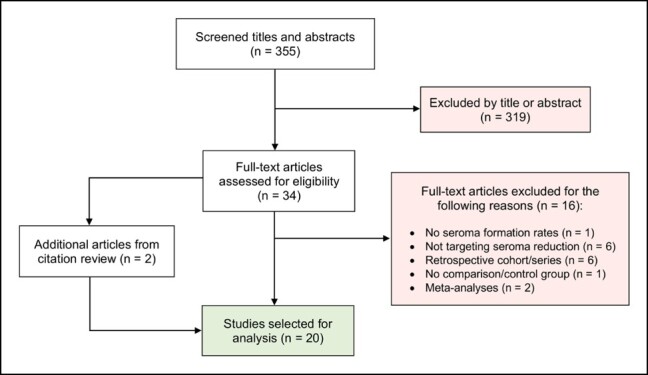
Flowchart describing our systematic literature review methodology.

A total of 40 articles met initial selection criteria; 2 additional articles were retrieved upon citation review of meta-analyses, and 22 articles were excluded upon full-text review. Ultimately, 20 articles were analyzed (Figure 1). The LOE for each article was determined according to the American Society of Plastic Surgeons (ASPS) Levels of Evidence Rating Scale for Prognostic/Risk Studies.^19^ The following data were extracted: study design, study period, number of patients, age, BMI, weight of resected tissue specimen (g), smoking status, proportion of females, technique(s) under investigation, seroma occurrence rate, methodology of diagnosing seroma, and follow-up duration. To compare seroma occurrence rates between techniques, we defined the “seroma occurrence ratio” as the percentage of seroma events in the interventional group divided by that in the control group, the definition of which varied due to inherent heterogeneity.

Microsoft Excel (Microsoft, Redmond, WA) was used to perform *χ*^2^ tests, Fisher's exact tests, and comparison of means using nonparametric, 2-tailed, 2-sample *t*-tests. Yates's *χ*^2^ tests were used for event counts (seroma or no seroma) less than 5. Interventions demonstrating 0% seroma rates were considered to have insufficient sample sizes for statistical analysis. Statistical significance was determined by *P*-values <.05. Meta-analysis of studies comparing standard drains and alternative techniques was performed using Review Manager (RevMan) Version 5.4 (Cochrane, London, England).^20^ For the meta-analysis, the primary outcome was clinical seroma occurrence reported as risk ratios with 95% CIs; estimates were pooled using the Mantel–Haenszel random-effects method. Heterogeneity was quantitatively assessed using the *I*^2^ statistic.

## RESULTS

### Study Characteristics

**Figure 2.**
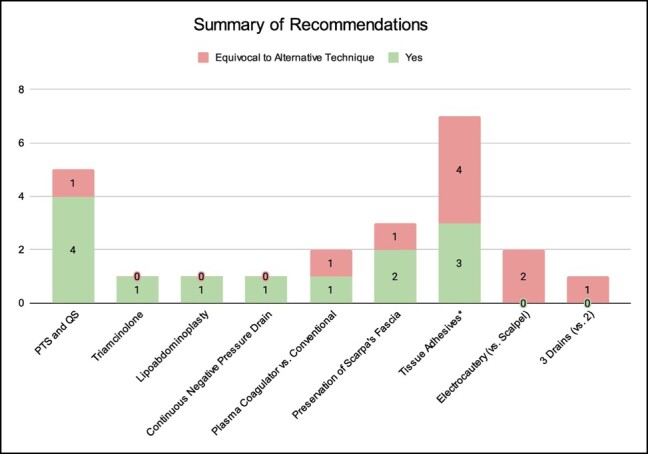
Summary of recommendations on interventions included in this review. The number of studies making each recommendation is reported.

Study designs included RCTs (*n* = 10), prospective cohort studies (*n* = 2), prospective comparative studies (*n* = 7), and retrospective randomized studies (*n* = 1). The year of publication ranged from 2007 to 2023, with 1 paper published prior to 2010. The average ASPS LOE was 1.1 ± 0.3. The average number of patients was 60, ranging from 20 to 160. Patients were followed postoperatively for a period ranging from 2 weeks to 20 months. They were assessed for seroma formation either clinically (*n* = 7), with ultrasound (*n* = 3), or both (*n* = 6); 4 studies did not specify (Table 1, Figure 2).

**Table 1.** Summary of Data Collected From the Selected Articles

Reference	Country of study	Year of publication	Study design	ASPS level of evidence	Study period	Number of patients	Technique under investigation	Follow-up	Method of diagnosis
Andrades et al (2007)^10^	Chile	2007	Prospective RCT	1	Jan 2003 to Jan 2005	55	PTS ± drains	6 mos	Clinical and US
Anker et al (2021)^11^	Germany	2021	RCT	1	Aug 2017 to Aug 2019	56	Triamcinolone injections into wound cavity	4 wks	US
Bercial et al (2012)^21^	Brazil	2011	Randomized prospective cohort	1	Mar 2008 to Oct 2008	43	Drains vs QS vs fibrin sealant	1 mo	US
Bromley et al (2018)^22^	Brazil	2018	Prospective RCT	1	Jan 2017 to Dec 2017	63	Number of PTS	6 mos	Clinical and US (≥ 50 cc)
Costa-Ferreira et al (2013)^23^	Portugal	2013	Prospective RCT	1	Aug 2009 to Feb 2011	160	Preservation of Scarpa’s fascia	6 mos	Clinical
Di Martino et al (2010)^24^	Brazil	2010	Prospective comparative study	1	Jan 2006 to Mar 2008	58	QS vs lipoabdominoplasty	2–3 wks	Clinical and US.
Hersant et al (2016)^25^	France	2016	Prospective cohort study	2	n/s	24	Autologous PRP glue	12 mos	n/s
Iannelli et al (2010)^7^	France	2010	Prospective RCT	1	Sept 2005 to Aug 2008	60	PlasmaJet system coagulator vs monopolar electrocautery	3 mos	Clinical
Inforzato et al (2020)^26^	Brazil	2020	Prospective randomized comparative study	1	May 2014 to May 2015	42	Preservation of Scarpa's fascia	3 wks	Clinical and US
Jiang et al (2019)^27^	China	2019	Prospective RCT	1	Feb 2010 to Apr 2014	58	Continuous negative pressure drain	n/s	n/s
Koller and Hintringer (2012)^28^	Austria	2012	Prospective comparative study	1	Feb 2008 to Feb 2010	50	Preservation of Scarpa’s fascia	6 mos	Clinical and US
Mabrouk et al (2013)^29^	Egypt	2013	Prospective RCT	1	n/s (3 years)	60	Fibrin glue	3 wks	US
Marsh et al (2014)^30^	United Kingdom	2014	Prospective RCT	1	n/s	102	Scalpel vs electrocautery for dissection	3 mos	Clinical
Pilone et al (2014)^32^	Italy	2014	Prospective RCT	1	Sept 2011 to Jul 2012	30	Fibrin sealant	2 wks	Clinical and US
Pisco et al (2019)^32^	Portugal	2019	Prospective comparative study	1	Sept 2016 to Mar 2019	73	3 vs 2 closed-suction drains	3 mos	Clinical
Schettino et al (2012)^33^	Brazil	2012	Prospective comparative study	1	n/s	40	Fibrin Glue (PPP)	≥3 mos	n/s
Schlosshauer et al (2019)^34^	Germany	2019	Retrospective randomized study	2	Jan 2017 to Dec 2018	52	Monopolar electrocautery vs PEAK PlasmaBlade	n/s	n/s
Spring (2018)^35^	USA	2018	Prospective comparative study	1	Jun 2016 to Feb 2017	20	PTS vs TissuGlu surgical adhesive	13–20 mos	Clinical
Valença-Filipe et al (2015)^36^	Portugal	2015	Prospective comparative study	1	Jan 2009 to Dec 2011	119	Scalpel vs electrocautery for dissection	6 mos	Clinical
Walgenbach et al (2012)^17^	Germany	2012	Prospective RCT	1	n/s	40	TissuGlu surgical adhesive	3 mos	Clinical

n/s, not specified; PPP, platelet-poor plasma; PRP, platelet-rich plasma; PTS, progressive tension suture; QS, quilting suture; RCT, randomized controlled trial; US, ultrasound.

**Figure 3.**
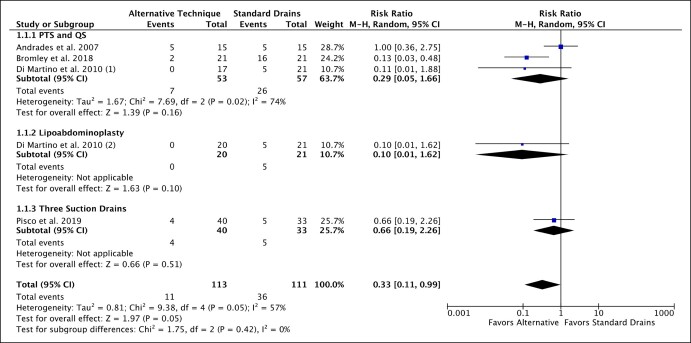
Forest plot of the meta-analysis comparing seroma rates of alternative techniques to those of standard drains.

A qualitative assessment of seroma reduction techniques is summarized in Table 2. The most frequently studied technique was utilizing tissue adhesives (*n* = 7), which includes fibrin sealants, fibrin glue, platelet-rich plasma (PRP), and other adhesives (TissuGlu, Oplotnica, Slovenia). Other techniques included PTS and QS (*n* = 5), preservation of Scarpa's fascia (*n* = 3), using electrocautery rather than scalpel for dissection (*n* = 2), triamcinolone injections (*n* = 1), adjunctive liposuction (ie, lipoabdominoplasty, *n* = 1), using plasma coagulation instead of conventional monopolar electrocautery (PEAK PlasmaBlade [Minneapolis, MN] or PlasmaJet [Atlanta, GA]; *n* = 2), applying continuous negative pressure drains postoperatively (*n* = 1), and increasing the number of drains (*n* = 1). The meta-analysis included 4 studies, of which the interventions were PTS, QS, lipoabdominoplasty, and using 3 drains; the control group for all of these was conventional abdominoplasty with placement of 2 drains (Figure 3).

**Table 2.** Summary of Techniques Other Than Standard Suction Drains Used by the Selected Studies to Prevent Seroma Occurrence Following Abdominoplasty

Technique/intervention	No. of studies	No. of involved patients	Demonstrated clear benefit?	Qualitative recommendation	Statistical significance
Tissue adhesives^a^	7	101	Yes (3); No (4)	Mixed	Yes
PTS and QS	5	99	Yes (4); No (1)	Yes	Yes
Preservation of Scarpa’s fascia	3	126	Yes (2); No (1)	Mixed	Yes
Electrocautery (vs scalpel)	2	138	Yes (0); No (2)	No	Equivocal
Plasma coagulator vs conventional electrocautery	2	57	Yes (1); No (1)	Mixed	Insufficient data
Triamcinolone	1	20	Yes (1); No (0)	Yes	Insufficient data
3 Drains (vs 2)	1	40	Yes (0); No (1)	No	Equivocal
Lipoabdominoplasty	1	20	Yes (1); No (0)	Yes	Insufficient data
Continuous negative pressure drain	1	29	Yes (1); No (0)	Yes	Equivocal

The number of patients undergoing each specified intervention (excluding patients in comparison/control groups) was recorded. PTS, progressive tension suture; QS, quilting suture. ^a^Includes fibrin sealant, fibrin glue, platelet-rich plasma, and TissuGlu).

### Patient Characteristics

This review included 1205 patients undergoing abdominoplasty (Table 1). Of these, 537 underwent conventional surgery, while 668 underwent adjunctive interventions to reduce seroma occurrence. The meta-analysis included 203 patients, with 113 and 90 patients in the intervention and control groups, respectively (Table 3). The mean age was 40 ± 6 years (range, 35-48 years), mean BMI was 25.6 ± 2.1 kg/m^2^ (range, 23.7-27.9 kg/m^2^), and mean resected weight was 765 ± 322 g (range, 589-2326 g). There was no statistically significant difference in the percentage of females (96% vs 93%, *P* = .4833) and smokers (15% vs 13%, *P* = .7293) between the interventional and control groups, respectively. There was also no significant difference in age (*P* = .5587), BMI (*P* = .7610), and resected weight (*P* = .9982) between the groups.

**Table 3.** Summary of Characteristics of Patients Included in the Meta-Analysis

	Alternative technique	Standard drains	*P*-value
No. of patients	113	90	NA
Age (years)	40 ± 5	41 ± 7	.5587
BMI (kg/m^2^)	25.64 ± 2.19	25.55 ± 1.96	.761
Resected weight (g)	764.9 ± 353.7	764.8 ± 277.0	.9982
% Smoking	15% (17/113)	13% (12/90)	.7293
% Female	96% (108/113)	93% (84/90)	.4833

NA, not applicable.

### Seroma Rate

The use of PTS and QS showed the greatest amount of data supporting a statistically significant reduction in seroma, with 4 of 5 studies clearly demonstrating a beneficial effect compared with their respective control groups (*P* < .001; Figure 2, Table 2). The seroma occurrence ratio was 0.306 (*P* < .001; Table 4), further confirming their efficacy.

**Table 4.** Seroma Occurrence Ratios for Each Technique

Technique	Seroma rate in intervention group	Seroma rate in control group	Seroma occurrence ratio	*P*-value
PTS and QS	14/99	31/67	0.31	<.00001
Preservation of Scarpa's fascia	5/126	22/126	0.23	<.001
Tissue adhesive	11/101	27/93	0.37	<.001
Continuous negative pressure drain	1/29	6/29	0.16	.1069
Electrocautery (vs Scalpel)	25/169	10/112	1.67	.1467
3 Drains (vs 2)	4/40	5/33	0.66	.7576
Lipoabdominoplasty	0/20	5/21	Insufficient sample size	
Plasma coagulator vs conventional electrocautery	0/57	5/55	Insufficient sample size	

Studies reporting seroma volume only were excluded. The seroma occurrence ratio was defined as the percentage of seroma events in the interventional group divided by that in the control group.

Studies examining tissue adhesives (fibrin sealants, fibrin glue, PRP, and TissuGlu) were the most numerous (*n* = 7) but revealed mixed results with regards to seroma reduction (3 studies demonstrated a significant benefit, but 4 studies did not; Figure 2). However, seroma occurrence ratio of tissue adhesives compared with their respective control groups was 0.375 (*P* < .01; Table 4), revealing a significantly lower seroma incidence. Similarly, among the 3 studies examining preservation of Scarpa's fascia, 2 demonstrated a benefit and 1 showed equivocal results (Figure 2). The seroma occurrence ratio for this technique was 0.229 (*P* < .001, Table 4), indicating a statistically significant reduction.

Other interventions such as continuous negative pressure drains, plasma coagulators (as opposed to monopolar electrocautery), lipoabdominoplasty (instead of conventional abdominoplasty), or triamcinolone injections all demonstrated a qualitative reduction of seroma rates (Figure 2), but statistical analysis did not support recommending these techniques (Table 4). Performing lipoabdominoplasty yielded a seroma rate of 0% compared with the control group rate of 23.8% with an insufficient sample size for statistical testing. Similarly, the use of plasma coagulators had a seroma occurrence ratio of 0.0 (control group rate: 9.1%), and statistical testing could not be performed. Continuous negative pressure drains showed equivocal results in seroma occurrence (seroma occurrence ratio of 0.164, *P* = .1069); however, these interventions had limited data supporting their benefit, given that only 1 study per intervention had met selection criteria. The publication on triamcinolone injections—a newer intervention for seroma reduction in abdominoplasty—reported average seroma volumes rather than proportions, and thus no occurrence ratio was calculated.

Two interventions—using electrocautery instead of scalpels and using more drains (3 vs 2)—did not show a qualitative benefit in reducing seroma formation (Figure 2). The seroma occurrence ratio for electrocautery use was 1.67 (*P* = .1467), indicating no significant difference in seroma formation rates. The study examining 3 drains (*n* = 73) revealed no significant difference in seroma rates (seroma occurrence ratio of 0.658, *P* = .7576).

### Meta-Analysis

The total number of patients was 113 for alternative techniques and 111 for standard drains, because 1 study^24^ contained 2 separate cohorts eligible for analysis, and the control group from this study was counted twice (Figure 3). As mentioned above, the total number of unique patients was 203 (Table 3). Three studies comparing PTS and QS to drains in abdominoplasties were meta-analyzed to reveal a risk ratio of 0.29 (95% CI, 0.05-1.66, *I*^2^ = 74%; Figure 3). Analysis of lipoabdominoplasty vs conventional drain use revealed a risk ratio of 0.10 (95% CI, 0.01-1.62) lacking statistical significance; however, only 1 study (*n* = 1) was included. Finally, analysis of 3 suction drains revealed a risk ratio of 0.66 (95% CI, 0.19-2.26), demonstrating no significant reduction in seroma formation. Although none of the individual 3 interventions examined in the meta-analysis demonstrated efficacy in reducing seroma formation, the overall pooled analysis demonstrated a statistically significant risk ratio of 0.33 (95% CI, 0.11-0.99, *I*^2^ = 57%) compared with the standard approach of placing 2 closed-suction drains.

## DISCUSSION

Seromas are one of the most common complications following abdominoplasty. The current review highlights recently published seroma reduction interventions with high-quality study designs. All of the selected articles were published within the past 2 decades, underscoring the recency of these techniques and indicating that many are still under investigation (Table 1). By exclusively evaluating papers with a high LOE (average grade 1.1 ± 0.3; Table 1)—with a majority of the selected publications comprised of RCTs and prospective comparative studies—we provide an expanded and contemporary overview of strong evidence-based recommendations, regarding the use of various methods to minimize seroma formation following abdominoplasty. Interestingly, in agreement with Seretis et al,^18^ we found that compared with conventional abdominoplasty, seroma rates can be reliably reduced by certain surgical techniques, which will be discussed in further detail in the following paragraphs.

Overall, 9 categories of techniques were analyzed (Table 1, Figure 2). The majority of available data pertains to tissue adhesives followed by PTS, QS, preservation of Scarpa's fascia, electrocautery compared with scalpels for dissection, and plasma coagulators compared with conventional monopolar electrocautery. The remaining techniques—triamcinolone injections,^11^ lipoabdominoplasty,^24^ continuous negative pressure drains,^27^ and using 3 rather than 2 closed-suction drains^32^—have only 1 high-quality publication each. While there is limited data for these techniques, the existence of high-level evidence suggests that they are already under close investigation. Furthermore, these techniques, with the exception of using 3 drains, qualitatively demonstrated potential benefit in reducing seroma formation. Given their promising results but acknowledged limitations due to small study numbers, further investigations are warranted to arrive at more definitive conclusions for these techniques.

Qualitative analysis of each technique revealed additional insight (Table 2). The technique with the least ambiguity in efficacy is the use of PTS or QS, which has demonstrated consistently positive outcomes in abdominoplasty (Table 2).^10,21,22,24^ Among the 5 PTS/QS studies, all but 1 study^35^ demonstrated a clear advantage of utilizing PTS or QS over other techniques to mitigate seroma formation. Furthermore, although 1 study showed no difference in PTS compared with a lysine-derived urethane surgical adhesive (TissuGlu), it should still be noted that both groups achieved a 0% seroma rate in a small cohort of 10 patients each, and PTS was still considered efficacious.^35^ The other 2 categories that demonstrated a statistically significant difference in seroma formation rates were the use of tissue adhesives and preservation of Scarpa's fascia. While these techniques are well-reinforced,^22,29-32,35^ there is conflicting evidence regarding their definitive benefit,^17,21,25,26,33,^ and there may not yet be enough data to draw a conclusion at the time of this review. Additionally, among the studies that demonstrated a substantial benefit, only 1 study^23^ reported a formal power analysis, bringing into question the statistical reliability of the conclusions presented in these publications despite their high ASPS-rated LOE.

Two techniques that have not demonstrated a significant benefit in seroma reduction and had equivocal statistical differences were the use of electrocautery (vs scalpel) for dissection^30,36^ and using 3 drains instead of 2 drains^32^ (Table 2). Given these findings, it is likely that other factors and methods are more important in influencing seroma occurrence rate following abdominoplasty. Notably, Pisco et al^32^ examined the use of 3 drains in the context of Scarpa's fascia preservation, which alone has already been demonstrated to reduce seroma formation and may have confounded the study's conclusions.^37^ Looking more closely at the method of dissection, while electrocautery may provide enhanced intraoperative control of bleeding, using a scalpel is thought to preserve overall tissue health by minimizing the amount of thermal injury and resultant inflammation and fluid accumulation.^38,39^ With regards to abdominoplasty, utilizing scalpel dissection may be more beneficial for seroma reduction when compared with electrocautery,^36^ but the evidence remains mixed overall and may depend on the level of each surgeon's experience.^30^ Regardless, when deciding between scalpel or electrocautery for surgical dissection of such a large tissue flap, factors other than seroma formation, such as hematoma risk, bleeding risk, and total operating time, must be carefully considered.

Two studies compared the rate of seroma formation following electrocautery and the use of plasma coagulators in abdominoplasty.^7,34^ Although plasma coagulators (ie, PEAK PlasmaBlade or PlasmaJet) yielded a promising seroma formation rate of 0%, the 2 studies reported a similarly low seroma rate in the electrocautery group, thereby questioning the specific benefit of using plasma coagulators in this context. Overall, these 2 studies had insufficient data to perform an appropriate statistical analysis, warranting future studies before arriving at an evidence-based recommendation. Similarly, although triamcinolone injections^11^ and lipoabdominoplasty^24^ demonstrate promising initial results in reducing seroma formation rates, consistency across multiple studies and larger sample sizes are required to achieve a more meaningful conclusion. Notably, the 1 lipoabdominoplasty paper included in this review^24^ recorded a mean volume of 1327 cc of lipoaspirate after infiltration using a superwet technique. The authors did not specify the method of liposuction, but there is also currently no strong evidence, suggesting increased seroma rates using ultrasound-assisted liposuction compared with power-assisted liposuction. Although higher lipoaspirate volumes may be associated with increased seroma rates, the overall evidence is mixed, partly due to variations in abdominoplasty techniques and degree of flap undermining, the latter of which likely plays a more direct role in determining seroma risk.^40^ In fact, a recent paper on surgical trends highlighted lower complication rates despite increased utilization of abdominal flap liposuction among plastic surgeons; the authors also partially attribute the decreased complication rates to diminished flap undermining,^41^ emphasizing the multifactorial nature of seromas and other complications. Similarly, although Dillerud^42^ published a higher rate of seroma formation when liposuction was combined with abdominoplasty compared with abdominoplasty alone, Gould et al^43^ showed no difference in seroma rates when liposuction was used with PTS, indicating that liposuction alone may not be as influential as once thought in increasing seroma risk.

A selective subgroup meta-analysis was performed to evaluate seroma occurrence in studies controlled by the use of standard drains (Figure 3). Most of the data in the meta-analysis was derived from studies on PTS and QS. Lipoabdominoplasty and the use of 3 drains—while included in the meta-analysis and demonstrate positive or equivocal outcomes—are limited by smaller sample sizes. Overall, our meta-analysis illustrates that compared with the standard placement of 2 drains, alternative techniques are likely more effective at reducing seroma occurrence, which is supported by another recent meta-analysis^18^ and motivates further research in this area.

Among the interventions examined in this systematic review, PTS and QS demonstrated the most reliable data supporting efficacy in reducing seroma formation rate. These studies were more numerous and consistently supported their ability to reduce seroma rates when applied in conjunction with drains.^10,21,22,24,35,^ While subgroup meta-analysis demonstrated that PTS and QS do not have a statistically significant effect on seroma prevention when compared with 2 standard drains, this result may be limited by the sample size of the meta-analysis. Nevertheless, the results of our analysis should be thoughtfully compared with those reported by other authors of publications with lower LOEs. Notably, this review examined PTS and QS together because they both involve suturing the raised abdominal flap to the underlying abdominal wall fascia to minimize dead space.^5,6^ However, the definitions of these 2 techniques may vary between authors, and the techniques themselves also likely vary between surgeons, which may influence overall outcomes.

**Figure 4.**
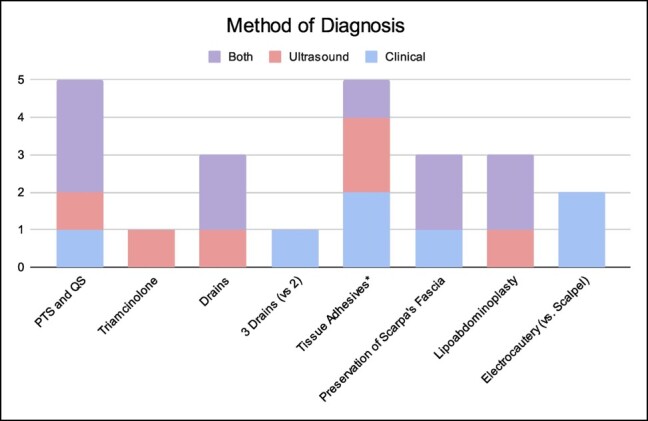
Method used to diagnose patients categorized by technique or intervention used.

There are several limitations to this systematic review. When performing the initial search, traditional abdominoplasty was not distinguished from Fleur-de-Lis abdominoplasty, although recent literature shows that this would be unlikely to influence seroma occurrence.^11^ The assignment of LOEs to each publication can be subject to interpretation bias and, consequently, selection bias; furthermore, Level 1 evidence as defined by ASPS does not necessarily correspond to high-quality evidence, and there are several factors omitted from the guidelines that can influence the quality of evidence regardless of study design.^44^ Additionally, as this study is a systematic review, there is inherent variability among patient populations and surgical techniques which may influence the efficacy of certain seroma prevention methods (variations in PTS techniques are a primary example). There was also a lack of standardization of the method used for diagnosing seroma that limited our analysis (Figure 4); we decided to examine clinically diagnosed seroma because of implied clinical significance, and the volume threshold for ultrasound-guided diagnosis may vary between practitioners. Finally, the number of eligible studies for the meta-analysis was limited by study designs: Not all studies had the same control group, but perhaps having 1 (ie, placement of 2 closed-suction drains) would be useful to enable a more robust meta-analysis in the future.

## CONCLUSIONS

This systematic review highlights multiple techniques used to reduce seroma occurrence in abdominoplasty that were investigated in recent high-quality literature. The beneficial effects of PTS and QS, tissue adhesives, and preservation of Scarpa's fascia are reinforced by high-quality evidence that is relatively consistent across multiple sources. Other potentially beneficial techniques such as triamcinolone injections, adjunctive liposuction, continuous negative pressure drains, and the use of plasma coagulators (vs conventional electrocautery) warrant further discussion and research in order to arrive at a more definitive consensus. Notably, there is a difference between qualitative recommendations and statistically significant evidence that should be considered prior to integrating new techniques into practice; furthermore, establishing standardized control groups across multiple studies would afford more meaningful pooled comparisons. Overall, we suggest future randomized comparative studies of the aforementioned seroma prevention methods under investigation to fully ascertain their efficacy following abdominoplasty.
